# Early Postnatal Androgenic Activity Inhibition Produces Differential Long‐Term Alterations in Anxiety‐Like Behavior in Young Adult Male and Female Wistar Rats

**DOI:** 10.1002/dev.70211

**Published:** 2026-10-15

**Authors:** Ulises Primo, Jose Manuel Fernández‐García, Rocío García‐Úbeda, Paloma Collado, Beatriz Carrillo, Helena Pinos

**Affiliations:** ^1^ Department of Psychobiology National University of Distance Education (UNED) Madrid Spain; ^2^ University Institute of Research‐UNED‐Institute of Health Carlos III (IMIENS) Madrid Spain

**Keywords:** androgens, anxiety‐like behavior, development, elevated plus maze, open field test, sex differences

## Abstract

Sex differences in anxiety‐like behavior are influenced by gonadal steroids, yet the contribution of early postnatal androgen signaling and testosterone metabolic pathways to the long‐term organization of these behaviors remains unclear. Wistar male and female rats received daily s.c. injections from postnatal day (P) 1 to P5 of vehicle, flutamide, letrozole, or finasteride. In young adult animals, locomotor activity and anxiety‐like behavior were evaluated via the open field test (OFT) and elevated plus maze (EPM). Females showed higher locomotor activity than males in both tests. Control females showed a less anxiety‐like profile than control males in the EPM. Flutamide treatment abolished sex differences in EPM open‐arm anxiety‐related measures and increased the number of freezing episodes and their duration in the OFT. However, finasteride treatment preserved the sex differences observed in the control groups in anxiety‐like measures. Finally, aromatase inhibition abolished sex differences in anxiety‐like behavior and induced an anxiolytic‐like effect in males, as indicated by increased open‐arm entries relative to control males. These findings indicate that androgen‐related pathways during early postnatal life shape adult anxiety‐like behavior in a sex‐dependent and pathway‐dependent manner and highlight aromatization as a key mechanism in the organization of EPM behavioral outcomes in males.

## 1 Introduction

Androgens are increasingly recognized for their effects on cognitive performance and emotional regulation in both human and animal models (Frye and Selinga 2001; Aspesi et al. 2025). Testosterone (T) typically activates the androgen receptor (AR) but can also indirectly activate estrogen receptor (ER)α or ERβ through its metabolites. T can be converted to estradiol (E) by the enzyme P450 aromatase or its reduced metabolite dihydrotestosterone (DHT) by the enzyme 5α‐reductase. DHT acts on the AR, but it can also act through ERβ (Handa et al. 2008) via one of its metabolites, 5α‐androstane‐3β,17β‐diol (3β‐diol). In animal models, it has been demonstrated that T has differential effects on adult males’ and females’ expression of anxiety‐like behaviors. Numerous experiments have reported sexual differences in anxiety‐like behavior in adult male and female rats, indicating that male rodents exhibit higher levels of anxiety‐like behavior than females (Zimmerber and Farley 1993; Leret et al. 1994; Brand and Slob 1988; Brown et al. 2015; Borbélyová et al. 2014, 2016). It has been proposed that endogenous T levels determine lower anxiety‐like behavior, and the age‐related decline in androgen production has been associated with increased anxiety‐like behavior in aging male rats (Domonkos et al. 2017). It has been shown that administration of T or DHT in adult male rats reduces anxiety‐like behaviors, as measured by the elevated plus maze (EPM) and open field test (OFT) (Aikey et al. 2002; Frye et al. 2008). This anxiolytic effect has been reported in both acute (Aikey et al. 2002; Frye and Edinger 2004) and chronic administration via ERs (Bitran et al. 1993; Osborne et al. 2009). Some studies have suggested that T exerts anxiolytic effects via aromatization (Aikey et al. 2002; Edinger and Frye 2004, 2006; Meng et al. 2011; Borbélyová et al. 2016). However, in females, evidence of the effect of T on anxiety‐like behavior in adulthood is scarce, but it has been demonstrated that continuous DHT administration from puberty, or 1‐week DHT exposure in adults, produces an increase in anxiety‐like behavior in females (Feng et al. 2011). Overall, the research seems to indicate that the sex‐specific effect of T on anxiety‐like behaviors in adult rats might be related to sex differences in estrogen and androgen signaling in the brains.

Early development and puberty are critical periods during which the body and brain can be influenced by both the organizational and activational effects of gonadal hormones, which determine sexual dimorphism in different physiological and behavioral processes, such as feeding or reproduction. Regarding the former, androgenic activity inhibition during the first 5 days of life induces alterations in body weight in adult females but not in adult males and differentially affects the amount of visceral fat in both males and females depending on which testosterone metabolic pathway is inhibited (Carrillo et al. 2024). Moreover, inhibition of aromatization, or reduction, influenced the hypothalamic expression of orexigenic peptides and ARs or ERs differentially in males and females (Carrillo et al. 2024; García‐Úbeda et al. 2025). Furthermore, androgenic and estrogenic activity during the first days of life also organize sexual differentiation and reproductive behaviors, as well as the structures that regulate them. This has been demonstrated for both the vomeronasal system and the preoptic area (Gorski 1993; Gorski et al. 1980; Guillamon and Segovia 1997). This organizational effect has also been shown in other emotional behaviors such as anxiety. Prenatal or perinatal exposure to T or altering T metabolism influences anxiety‐like behavior in both adult male and female rats. Exposure to elevated levels of T during pregnancy or prenatal inhibition of T aromatization has been reported to increase anxiety‐like behaviors related to alterations in the limbic system, especially in adult female offspring (Ordyan et al. 2007; Hu et al. 2015; Petrovic et al. 2019). Data obtained in males have shown that postnatal or prepubertal castration results in higher locomotor activity, more time spent in the center zone (CZ) of the OFT, and more time in the open arms of EPM, as well as a higher number of novel object visits in adult rats, compared with sham‐operated counterparts (LUCION et al. 1996; Zuloaga et al. 2011; Brown et al. 2015). Finally, evidence suggests that females receiving neonatal testosterone propionate treatment exhibited either male‐like OFT performance or an intermediate emotional phenotype following chronic stress.

This suggests that early introduction of androgens can masculinize exploratory and emotional responses in females (Stewart et al. 1975; Seney et al. 2012). As regards the effects of pubertal androgens on anxiety‐like behavior, research using animal models of adolescence to examine the ontogeny of anxiety has yielded conflicting results. In rats, it has been reported that adolescent males exhibit higher anxiety levels compared to adults when tested in a light/dark box, OFT, or EPM (Slawecki 2005; Doremus et al. 2003; Doremus‐Fitzwater et al. 2009; Lynn and Brown 2010). However, other authors have reported lower anxiety levels during adolescence, or late adolescence, when compared to younger or older animals (Imhof et al. 1993; Macrì et al. 2002), while some have found no age‐related differences in baseline anxiety levels between pubertal and adult animals (Varlinskaya and Spear 2002; Slawecki and Roth 2004). In the EPM, adolescent female rats have been reported to exhibit more open arm activity than age‐matched male rats (Elliott et al. 2004; Lynn and Brown 2009), although other studies have shown that this sex difference does not emerge until early adulthood (Imhof et al. 1993; Estanislau and Morato 2006). However, the precise onset of sex differences in anxiety‐like behavior is still not known.

It is worth noting that most experimental approaches used to study the early postnatal influence of androgens on the introduction or maintenance of anxiety‐like behavior in rats involve permanent alterations in gonadal hormone signaling, such as castration, leading to altered response patterns characterized by masculinization or feminization of behaviors studied. However, the contribution of early postnatal androgen signaling and the main testosterone metabolic pathways, aromatization and 5α‐reduction, to the long‐term organization of these behaviors remains poorly understood. The present study evaluates whether a transient inhibition of androgenic activity during a possibly critical early postnatal period can induce long‐term effects on anxiety‐like behavior in postpubertal male and female rats, while also allowing the relative contributions of androgen signaling and these metabolic pathways to be brought to light. To this end, androgenic activity was markedly reduced by inhibiting the AR, or the enzymatic activity of P450 aromatase or 5α‐reductase, during the first 5 days of postnatal life, and performance in the OFT and EPM was assessed in young adult male and female rats.

## 2 Materials and Methods

### 2.1 Animals and Experimental Treatments

Special care was taken to ensure animal welfare and to minimize the number of animals used. Wistar male and female rats (Charles River, Lyon, France) were kept in an automatically controlled room, in standard conditions (12‐h light/12‐h dark cycle, lights on from 8:00 a.m. to 8:00 p.m.; 22°C ± 2°C; 55 ± 10% humidity), with water and food ad libitum (chow diet: 20% proteins, 5% fat, 55% carbohydrates, 3438 kcal/kg [Altromin, Lage, Germany]). Animal care and handling throughout the study were supervised and approved by the Local Institutional Bioethical Committee (UNED, Madrid). All experimental protocols were performed in compliance with “Guidelines for the Use of Animals in Neuroscience Research” by the Society for Neuroscience, the European Union regulation (Council Directives 86/609/EEC and 2010/63/EU), as well as the Spanish Government Directive (R.D. 53/2013).

For mating, one male and two females were placed in each cage for 1 week. All the rats were bred in the same week to ensure births within the shortest time frame possible. Once pregnancy was detected, females were transferred to individual plastic maternity cages with wood shavings provided for nesting. The 12 pregnant females were randomly assigned to four experimental conditions, ensuring that at least two dams that gave birth on the same day were allocated to each experimental condition (control, finasteride, flutamide, and letrozole). The day of birth was designated as postnatal day 1 (P1). On P1, pups were sexed, weighed, and subsequently redistributed among litters born within the same 24‐h period, standardizing each litter to 10 pups (five males and five females). From P1 to P5, all neonates received daily subcutaneous injections (volume of 0.01 mL) of one of the following treatments: (A) Vehicle (VH; sesame oil); (B) Flutamide (Flu; 25 mg/kg; F9397, Merck), a competitive AR antagonist that prevents the nuclear translocation of the receptor complex; (C) Finasteride (Fin; 5 mg/kg; NB‐48–0403–100MG, Quimigen), inhibitor of 5‐α‐reductase enzyme; and (D) Letrozole (Let; 1 mg/kg; L6545, Merck), an aromatase inhibitor. Doses were selected following previous literature and validated protocols (Fanaei et al. 2013, Yamada et al. 2015, Cataldi et al. 2018, Cheng et al. 2019, Lagunas et al. 2022, Carrillo et al. 2024, García‐Úbeda et al. 2025). Figure 1 shows the experimental design and molecular targets of the treatments. Accordingly, eight experimental groups were established: (1) control males (CM = 11); (2) control females (CF = 12); (3) flutamide‐treated males (FluM = 12); (4) flutamide‐treated females (FluF = 12); (5) finasteride‐treated males (FinM = 12); (6) finasteride‐treated females (FinF = 12); (7) letrozole‐treated males (LetM = 15); and (8) letrozole‐treated females (LetF = 15). After weaning, on P21, the rats were housed individually. Behavioral tests were conducted as described below, from P58 to P67, the period that coincides with late adolescence and young adulthood in rats (Lynn and Brown 2010; Vetter‐O'Hagen and Spear 2012).

**FIGURE 1:**
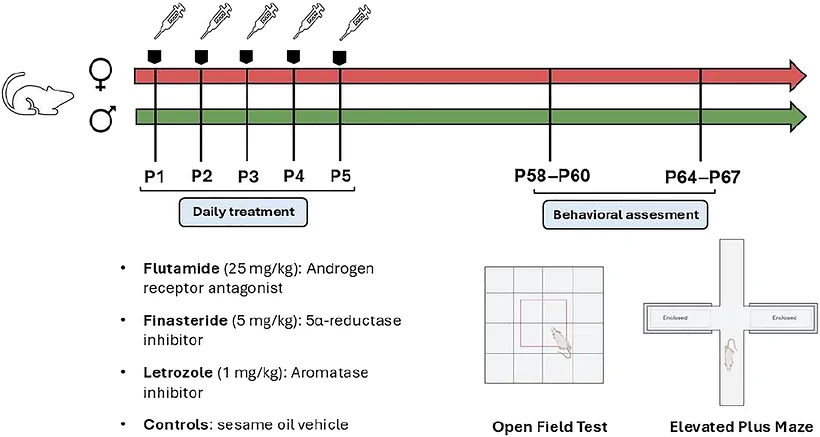
Experimental design and pharmacological targets. Male and female pups were treated during the first five postnatal days with flutamide, a competitive androgen receptor antagonist; finasteride, a 5α‐reductase inhibitor; or letrozole, an aromatase inhibitor. The open field test was conducted between P58 and P60, followed by the elevated plus maze between P64 and P67. Before the start of each test, all animals were handled for 3 days to habituate them to experimental manipulations.

### 2.2 Behavioral Tests

Anxiety‐like behavior and locomotor activity were assessed using two behavioral tests: OFT and EPM. All animals were first assessed in the OFT and subsequently in the EPM 1 week later. Within each test, the order of evaluation was counterbalanced across sex and treatment groups. In all cases, tests were conducted between 2:00 p.m. and 7:00 p.m. Prior to each test, the rats were handled 3 days before to habituate them to experimental manipulations. The apparatus was cleaned between subjects with a 1:1000 acetic acid solution to remove any odor traces.

#### 2.2.1 OFT

Between P58 and P60, animals were tested in the OFT. Each rat was allowed to freely explore in a novel arena (open field: 50 × 50 × 50 cm, made of methacrylate) for 5 min. The maze was divided into two regions: an inner zone (central square corresponding to 50% of the total area) and an outer zone (the peripheral area corresponding to the remaining 50%) (Seibenhener and Wooten 2015). All animals were initially placed in the center of the apparatus. Testing was performed under low‐light conditions, with approximately 80 lux measured at the center of the arena. Behavioral sessions were recorded using a digital camera, and videos were automatically analyzed using Any‐maze software (version 7.53, Stoelting Co., USA). Parameters measured included total distance traveled (cm), number of freezing episodes, freezing duration(s), number of entries into the inner zone, number of entries into the outer zone, percentage of time spent in the inner zone, and percentage of time spent in the outer zone.

#### 2.2.2 EPM

Between P64 and P67, rats were tested in the EPM (Walf and Frye 2007). The apparatus consisted of two opposing open arms (OA) (45 × 10 cm) and two closed arms (CA) (45 × 10 cm, with 50‐cm‐high walls) that extend from a central zone (10 × 10 cm), with a height of 65 cm above the floor. The EPM was made from methacrylate and expanded PVC. Animals were always placed in the central zone, facing the same enclosed arm at the beginning of the trial. The illumination was approximately 30 lux in the OA and 10 lux in the CA. The behavior in each experiment was monitored using a digital camera and automatically analyzed using Any‐maze software (version 7.53, Stoelting Co., USA). Parameters measured included total distance traveled (cm), number of freezing episodes, freezing duration(s), number of entries into OA, number of entries into the CZ, percentage of time spent in the OA, and percentage of time spent in the CZ.

#### 2.2.3 Statistical Analysis

Data from all animals were included in the final analysis, except for those whose data could not be properly recorded due to technical malfunctions. The data were analyzed using two‐way ANOVA with sex and treatment as factors. Post hoc comparisons between sexes within each treatment group were performed using Sidak's multiple comparisons test. Treatment effects within each sex were evaluated using Dunnett's multiple comparisons test, with each treated group compared with its respective sex‐matched control group. All statistical analyses were performed using R Statistical Software (version 4.5.3;) with a statistical significance of *p* < 0.05.

## 3 Results

### 3.1 Behavioral Test

#### 3.1.1 OFT

In the OFT, a significant main effect of sex was observed in locomotor activity (*F*_1, 88_ = 9.070; *p* = 0.003; *η*_p_^2^ = 0.093), with females covering a greater distance than males (Figure 2A). No significant main effect of treatment or sex × treatment interaction was found.

**FIGURE 2:**
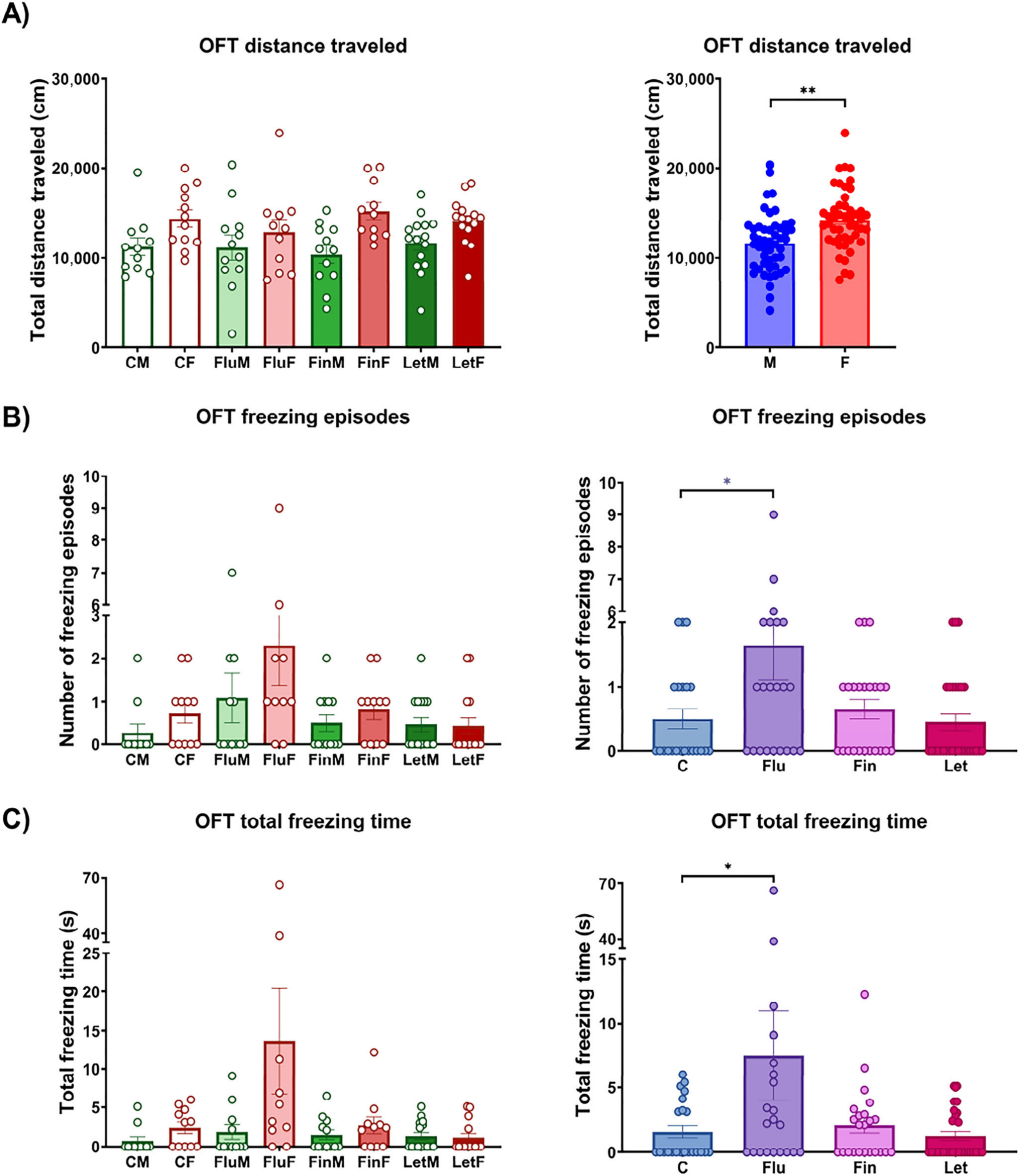
Neonatal androgen receptor blockade increased freezing behavior in the open field test (OFT). Bar charts show (A) total distance traveled, (B) the number of freezing episodes, and (C) total freezing time during the OFT. Left‐hand graphs show all eight experimental groups. Right‐hand graphs illustrate the main effect of sex on distance traveled, with treatment groups pooled (A), and treatment‐related differences in freezing behavior, with sexes pooled (B, C). Females traveled a greater distance than males, whereas flutamide‐treated animals exhibited more freezing episodes and longer total freezing times than control animals. Bars show mean ± SEM; dots represent individual animals. Breaks in the *y*‐axes in panels B and C improve visualization. **p < 0.01 for the main effect of sex; *p < 0.05 versus control for treatment comparisons. CM, control males; CF, control females; FluM, flutamide‐treated males; FluF, flutamide‐treated females; FinM, finasteride‐treated males; FinF, finasteride‐treated females; LetM, letrozole‐treated males; LetF, letrozole‐treated females; M, males; F, females; C, control; Flu, flutamide; Fin, finasteride; Let, letrozole.

Regarding anxiety‐related outcomes, a significant sex effect was detected in total freezing time (*F*_1, 88_ = 4.229; *p* = 0.043; *η*_p_^2^ = 0.046), with females displaying longer freezing times than males, irrespective of treatment. Moreover, significant main effects of treatment were detected for both the number of freezing episodes (*F*_3, 88_ = 4.419; *p* = 0.006; *η*_p_^2^ = 0.131) and total freezing time (*F*_3, 88_ = 4.770; *p* = 0.004; *η*_p_^2^ = 0.140). Post hoc comparisons showed that flutamide‐treated animals displayed more freezing episodes (*p* = 0.023; *η*_p_^2^ = 0.091) and longer total freezing times (*p* = 0.018; *η*_p_^2^ = 0.096) than vehicle‐treated animals, indicating an anxiogenic‐like effect of flutamide (Figure 2B,C). However, no significant sex × treatment interactions were detected for either freezing measure. No other significant effects were detected for entries nor for time spent in the inner or outer zones (Figure 3).

**FIGURE 3:**
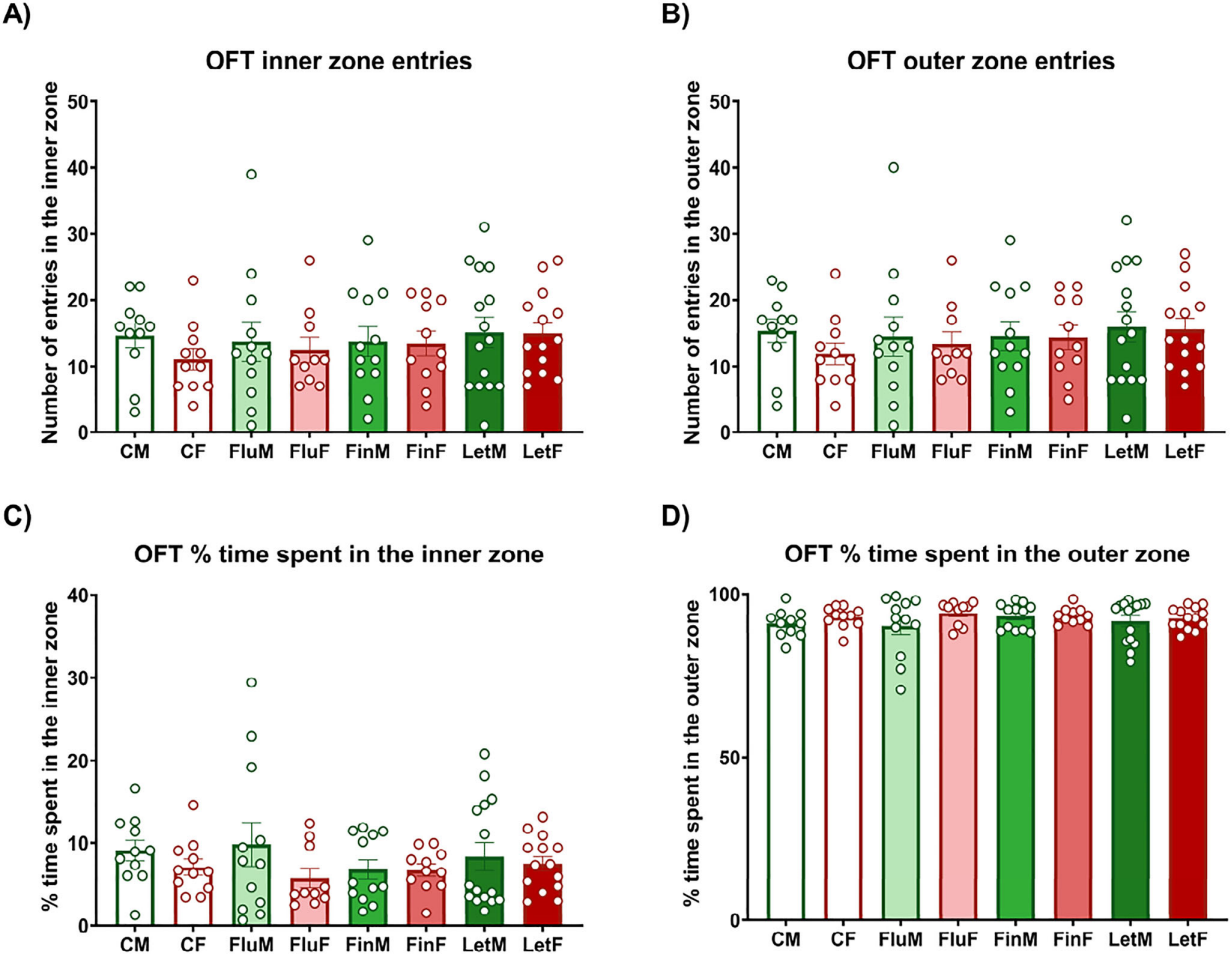
Entries into and percentage of time spent in the inner and outer zones of the open field test (OFT). Bar charts show the number of entries into the inner (A) and outer (B) zones, and the percentage of time spent in the inner (C) and outer (D) zones during the OFT. Bars show mean ± SEM; dots represent individual animals. CM, control males; CF, control females; FluM, flutamide‐treated males; FluF, flutamide‐treated females; FinM, finasteride‐treated males; FinF, finasteride‐treated females; LetM, letrozole‐treated males; LetF, letrozole‐treated females.

#### 3.1.2 EPM

Regarding locomotor activity in the EPM, a significant main effect of sex was detected (*F*_1, 88_ = 27.909; *p* < 0.001; *η*_p_^2^ = 0.241), with females covering a greater distance than males (Figure 4B). No significant main effect of treatment or sex × treatment interaction was detected.

**FIGURE 4:**
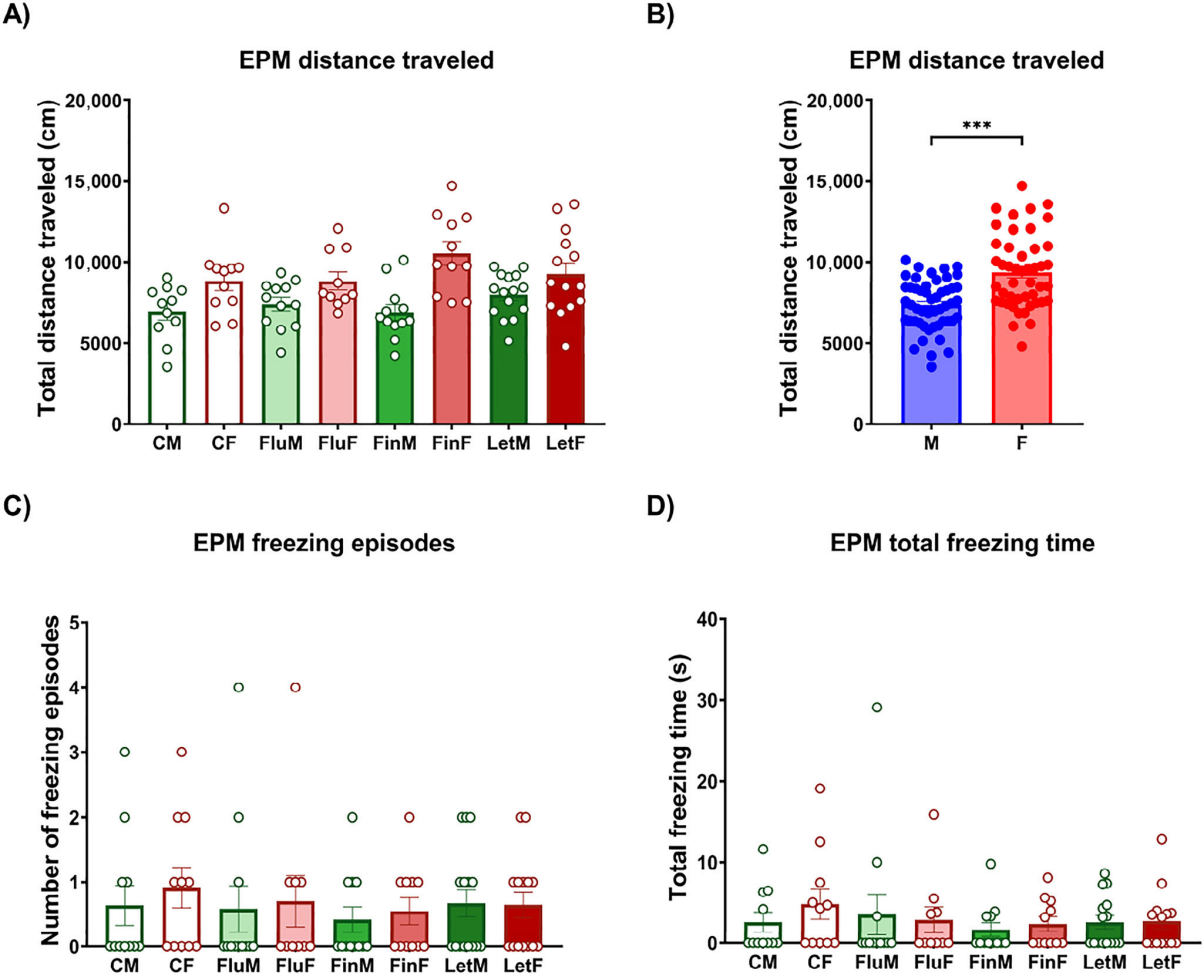
Locomotor activity and freezing behavior in the elevated plus maze (EPM). Bar charts show total distance traveled for all eight experimental groups (A) and for males and females with treatment groups pooled (B), the number of freezing episodes (C), and total freezing time (D) during the EPM. A significant main effect of sex was detected for distance traveled, with females covering a greater distance than males. Bars show mean ± SEM; dots represent individual animals. ***p < 0.001 for the main effect of sex. CM, control males; CF, control females; FluM, flutamide‐treated males; FluF, flutamide‐treated females; FinM, finasteride‐treated males; FinF, finasteride‐treated females; LetM, letrozole‐treated males; LetF, letrozole‐treated females; M, males; F, females.

As regards anxiety‐related parameters, two‐way ANOVA revealed significant main effects of sex on the number of CZ entries (*F*_1, 88_ = 7.421; *p* = 0.008; *η*_p_^2^ = 0.078), the number of OA entries (*F*_1, 88_ = 26.104; *p* < 0.001; *η*_p_^2^ = 0.229), and the percentage of time spent in the OA (*F*_1, 88_ = 15.414; *p* < 0.001; *η*_p_^2^ = 0.149). In addition, significant sex × treatment interactions were detected in the number of CZ entries (*F*_3, 88_ = 3.913; *p* = 0.011; *η*_p_^2^ = 0.118) and in the number of OA entries (*F*_3, 88_ = 3.032; *p* = 0.033; *η*_p_^2^ = 0.094). Post hoc analyses revealed that CF showed greater activity in the OA than CM (Figure 5A), as shown by more OA entries (*p* < 0.001; *η*_p_^2^ = 0.145). Moreover, CF showed a greater number of CZ entries than CM (*p* = 0.003; *η*_p_^2^ = 0.091). Neonatal exposure to flutamide or letrozole abolished the sexual dimorphism naturally observed in control animals in the number of OA entries (Figure 5A). Likewise, both treatments eliminated the significant differences found between control groups in the number of CZ entries. Furthermore, an anxiolytic effect was observed in the number of OA entries in letrozole‐treated males, with males exposed to this treatment exhibiting a higher number of entries than CM (*p* = 0.050; *η*_p_^2^ = 0.052). Moreover, CM entered fewer times in the CZ than FluM (Figure 5B; *p* = 0.035; *η*_p_^2^ = 0.087). No significant differences were detected between CM and FinM. No significant differences were detected between CF and any treated female group.

**FIGURE 5:**
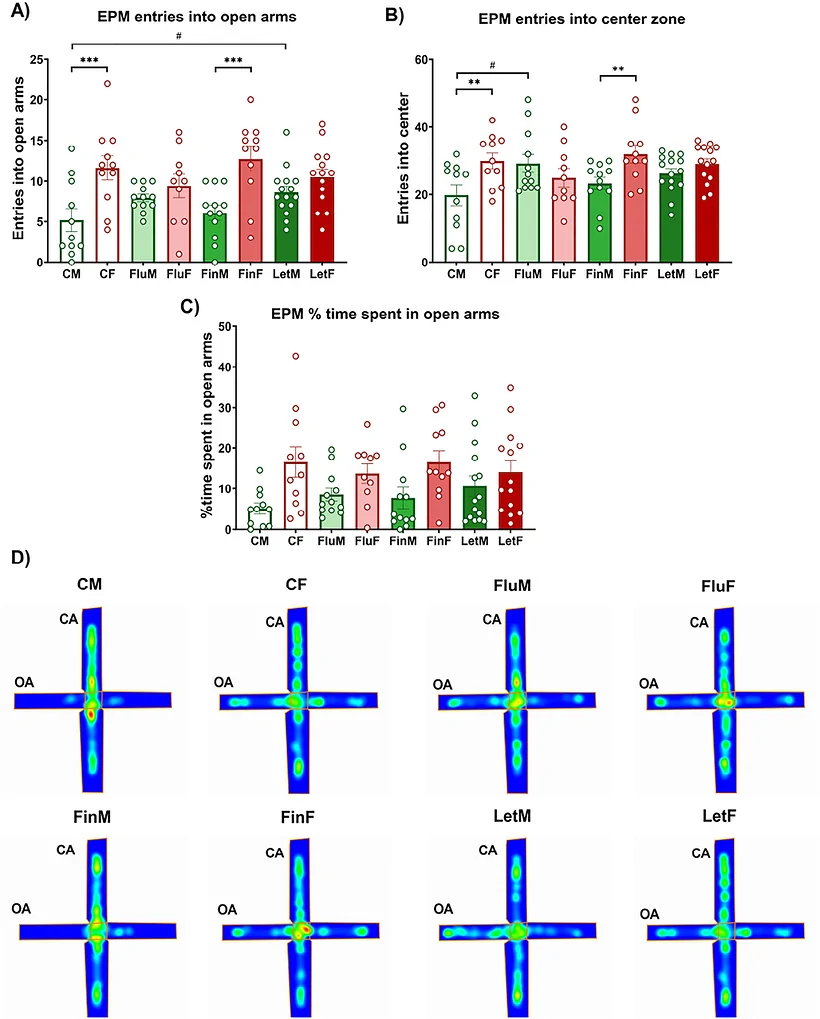
Neonatal androgen receptor blockade or aromatase inhibition abolished sex differences in open‐arm exploration, whereas neonatal aromatase inhibition increased entries into the open arms in males. Bar charts show anxiety‐related parameters in the elevated plus maze (EPM), including the number of entries into the open arms (OA) (A), the number of entries into the center zone (B), and the percentage of time spent in the OA (C). Females displayed more OA and center‐zone entries than males in the control and finasteride groups, whereas no significant sex differences in these measures were detected in the flutamide and letrozole groups. In males, neonatal letrozole treatment increased the number of entries into the OA, while flutamide treatment increased the number of center‐zone entries. (D) Representative heatmaps of spatial occupancy during the EPM session from one animal of each experimental group. Bars show mean ± SEM; dots represent individual animals. *p < 0.05, **p < 0.01, ***p < 0.001 (male vs. female within treatment); #*p* < 0.05 (vs. same‐sex control group). OA, open arms; CA, closed arms; CM, control males; CF, control females; FluM, flutamide males; FluF, flutamide females; FinM, finasteride males; FinF, finasteride females; LetM, letrozole males; LetF, letrozole females.

In finasteride groups, the sexual dimorphism observed in the OA entries was preserved (Figure 5A; *p* < 0.001; *η*_p_^2^ = 0.158). Similarly, this sex difference was also observed in the number of center entries (*p* = 0.012; *η*_p_^2^ = 0.075), with females entering the CZ more frequently than males (Figure 5B) in these groups.

No significant main effects or interactions were detected for freezing behavior in this behavioral test (Figure 4C,D).

## 4 Discussion

The primary findings of the present study indicate that blocking androgen signaling and the metabolic pathways of T during the first 5 postnatal days produces long‐term differential effects on anxiety‐like behaviors in male and female rats. However, these effects depend on the parameter measured and the metabolic pathway affected. Aromatase inhibition produced the most robust effects in males, inducing an anxiolytic‐like profile in males in the EPM, as shown by increased OA activity relative to control males, and abolishing sex differences in the long term. However, blocking AR increased the anxiety‐like profile seen in higher freezing behavior in the OFT in all animals. Yet, in the EPM, blocking this receptor abolished sex differences in anxiety‐like behavior, as well as increased the number of CZ entries in males.

In the present study, we employed the OFT and EPM to measure exploratory activity and anxiety‐like behavior, relying on the natural conflict between rats’ innate tendency to explore new environments and their aversion to open and elevated spaces (Pellow et al. 1985; Schmitt and Hiemke 1998; Walf and Frye 2007; Seibenhener and Wooten 2015; Gaspar et al. 2023; Bowman et al. 2025). In both tests, females showed greater locomotor activity than males, consistent with previous findings in both adult and late‐adolescent animals (Brand and Slob 1988; Zimmerber and Farley 1993; Leret et al. 1994; Bishnoi et al. 2020). AR blocking, inhibiting aromatization or reduction, did not affect this parameter, but Hodosy et al. (2012) showed an anxiolytic effect of AR blockage in intact males. This result is challenged by others who reported that neonatal flutamide exposure produced no significant anxiety changes in preadolescent males but increased depression‐like behavior (Zhang et al. 2010).

More interesting is the fact that in the current experiment, flutamide‐treated animals increased freezing behavior. With our experimental design, we have demonstrated that androgenic activity on AR during the first 5 days of life is crucial for male and female anxiety‐like behavior expression in young adulthood. We cannot provide an explanation for this finding at this time; however, it seems that inhibiting AR does not reduce anxiety‐like behavior in males per se but prevents or reverses androgen‐mediated anxiety in these animals. This suggests that early AR signaling is particularly important for establishing sexually differentiated behavioral patterns, as can be seen in EPM. This is consistent with the highly organized and multifactorial nature of behavioral sexual differentiation, which depends on hormonal influences across different critical periods, sex chromosome effects, and environmental factors (Arnold 2009). With our experimental design, we demonstrate that androgenic activity through the AR and aromatase activity during the first 5 days of life are crucial for the expression of sex differences in anxiety‐like behavior in young adulthood. These findings also stress the importance of including both sexes in developmental organization of anxiety‐like behavior studies, as analyses restricted to a single sex may fail to reveal alterations in sexually differentiated behavioral patterns.

The EPM and OFT are not entirely equivalent or interchangeable, as they differ in the type and intensity of threat posed, the motivational conflict elicited, the relative weight of exploration/locomotion versus avoidance, and their sensitivity to methodological and individual variables (Carola et al. 2002; Figueiredo Cerqueira et al. 2023; Himanshu et al. 2020; Liu et al. 2021). In the EPM, the anxiety component is generally considered more intense because animals are exposed not only to an open space but also to an elevated, unprotected height, thereby intensifying the conflict between exploring novel areas and seeking protection. Greater OA activity (entries and time) is considered an anxiolytic measure and is influenced by estradiol (Lund et al. 2005; Walf and Frye 2007; Figueiredo Cerqueira et al. 2023). However, this test is not considered an optimal tool for assessing locomotor activity (Walf and Frye 2007). In contrast, the OFT is typically administered in an enclosed space that facilitates thigmotaxis and rapid habituation, so factors more closely associated with pure anxiety may be strongly influenced by locomotor or exploratory activity (Ennaceur et al. 2006; Himanshu et al. 2020; Figueiredo Cerqueira et al. 2023). It must be mentioned that the classic parameters used in these tests may fail to capture more subtle behavioral patterns that emerge when applying more complex analytical approaches (Liu et al. 2021). Taken together, our findings suggest that early modulation of androgen signaling or testosterone‐related pathways shapes specific components of young adult anxiety‐like behavior in a sex‐ and pathway‐dependent manner, rather than producing uniform effects across parameters. With regard to anxiety‐like behavior assessed in the EPM, the activity in open arms is considered the primary endpoint (Walf and Frye 2007). In our study, CF exhibited more activity in the OA than CM, consistent with previous literature (Brand and Slob 1988; Ordyan et al. 2007; Johnson and File 1991; Scholl et al. 2019). Both AR and aromatase inhibition provoke an increase in entries in open arms in males compared to females, suggesting that aromatization activity during the first 5 days of life may contribute to the developmental organization of this behavior. This interpretation is further supported by the increased number of OA entries observed in males treated with the aromatase inhibitor relative to control males. However, it has been reported that AR inhibition with flutamide in the first 24 h after birth failed to demonstrate differences in anxiety‐like behavior in male rats (Zhang et al. 2010). In adult rats, testosterone acts upon AR to reduce anxiety‐like behavior, but the effect is not due to AR activation because DHT treatment does not reduce fear generalization (Carrier et al. 2015; Chen et al. 2014; Edinger and Frye 2006). Similarly, in rats with the testicular feminization mutation (Tfm) in which AR is partially or totally inhibited, locomotor behaviors did not differ between Tfm males and CM, suggesting that sex differences in activity in rats may be mediated by ER activation (Dalla et al. 2005; Zuloaga et al. 2011), mainly through activation of ERβ because administration of ERβ agonist (but not ERα) reduced anxiety‐like behavior in gonadectomized male and female rats as measured by the EPM and OFT (Kudwa et al. 2014; Lund et al. 2005).

An interesting increase in CZ entries in FluM compared with CM was also observed in our study. FluM displayed a distinct behavioral pattern characterized by greater exploration of the CZ, although this effect did not extend to open‐arm measures and therefore did not reach the broader anxiolytic‐like profile observed in LetM. This pattern is also apparent in the representative heatmaps, which show greater occupancy of the CZ, an area representing an intermediate level of aversiveness between the protected closed arms and the more anxiogenic open arms. Together, these findings suggest that the establishment of sex differences in this behavioral domain is particularly dependent on the conversion of testosterone to estradiol by aromatase. Nevertheless, the abolition of sex differences following AR blockade indicates that AR signaling also contributes to the developmental organization of these behavioral systems. Moreover, the absence of comparable effects following finasteride treatment, together with the effects observed after flutamide administration, suggests that testosterone itself, rather than DHT, may be the principal androgen acting through the AR during this critical developmental window.

In females, in contrast, there is no effect on anxiety‐like behavior in the EPM when androgenic activity is inhibited early after birth, indicating that, at least in females, other hormones like estrogens could organize this behavior. The different effects observed in the OFT and EPM may reflect the distinct characteristics and behavioral demands of each test. There are differences between the mechanisms underlying the establishment and maintenance of anxiety‐like behavior in males and females. This highlights the need to include both sexes in studies to gain a comprehensive understanding of this behavior.

Sex differences in anxiety‐like behavior related to the organizational and activational effects of gonadal hormones have been reported previously in the literature (for review, see Domonkos et al. 2018). According to the current hypothesis, gonadal steroids organize some brain circuits during sensitive prenatal and/or postnatal periods, thereby shaping later behavioral responses that are subsequently modulated by activational hormonal effects in adulthood. As we know, there are critical periods throughout life that are crucial for the organization of neuronal circuits and the pre‐programming of the activational effect of androgens. The period from P1 to P5 seems to be important for the organizational actions of androgens on energy metabolism, on the appropriate developmental pattern of the gonadal expression of gonadal receptors in the female hypothalamus, and on the neuroendocrine functions of the pituitary and adrenal glands (Carrillo et al. 2024; García‐Úbeda et al. 2025; Lagunas et al. 2022), indicating that, in rats, acute inhibition of androgenic activity during these critical periods permanently organizes hormones and their receptor systems in a sex‐specific manner.

Neonatal androgens have also been linked to the sex differences in anxiety‐like behavior. Excess testosterone during the gestational period has been associated with an increased risk of developing anxiety disorders in female offspring (Hu et al. 2015). Moreover, inhibition of aromatization in pregnant dams results in increased anxiety in both males and females (Ordyan et al. 2007; Pivina et al. 2007). There is no consensus in the literature regarding the effects of puberty on anxiety‐like behavior due to methodological differences in the stages of puberty tested. In rats, adolescence or puberty spans from P28 to P55, with early to mid‐adolescence spanning from P28 to P42, late adolescence from P43 to P55, and young adulthood from P65 to P69 (Lynn and Brown 2010; Vetter‐O'Hagen and Spear 2012). Moreover, the onset of puberty is different between females (P30–P32) and males (P35–P40). Taking this into account, during puberty, the most consistent finding is that females show lower anxiety‐like behavior than males. This difference appears to be age‐related since around P60, when rats are considered sexually mature, animals show an adult anxiety‐like behavior pattern (Masur et al. 1980; Imhaf et al. 1993; Johnston and File 1991; Lynn and Brown 2010), but not during early adolescence (Cassarrubea et al. 2025). Our results are in line with the above, indicating that sex differences in the pattern of anxiety‐like behavior in young adults depend on organizational/activational actions of gonadal steroids in both perinatal and pubertal periods, and suggesting that anxiety‐like behavior is dependent on consolidation of adult gonadal hormone patterns in each sex (Brown et al. 2015, LUCION et al. 1996, Lynd and Brown 2009; Petrovic et al. 2019, Renczés et al. 2020, Zimmerberg and Farley 1993). These findings support the premise that, in addition to the perinatal period, puberty is a critical period for behavioral and neurobiological maturation in both sexes.

Permanent inhibition of androgenic or estrogenic activity in males or females, respectively, alters the typical anxiety‐like behavioral response in adulthood (LUCION et al. 1996; Goel and Bale 2008; Zuloaga et al. 2011). It is worth noting that unlike a permanent suppression approach, the methodology employed in this study, an acute intervention finishing on the fifth day of life (P5), allows us to differentiate between the organizational and activational influences of gonadal steroids on anxiety. This is because the animals retain anatomically preserved gonads, allowing them to secrete hormones in accordance with normal ontogenetic development.

This model allows pubertal hormonal changes to occur in animals with intact gonads, so puberty may act on the developmental window and potentially contribute to compensatory neuroplastic processes following the neonatal manipulation. In addition, unlike permanent gonadectomy‐based approaches or genetic models with permanent disruption of androgen signaling, our transient pharmacological manipulation is restricted to an early postnatal period, thereby ensuring that adult hormonal function remains and eliminating severe side effects that might otherwise skew behavioral or neurological data. To our knowledge, our results are the first to evaluate the effects of early postnatal acute inhibition of androgenic activity on anxiety‐like behavior in young adulthood in both male and female rats, supporting the hypothesis that early gonadal hormones contribute to the organizational basis of sex differences in anxiety‐like behavior.

## 5 Conclusions

According to current experimental studies, androgenic activity early in life predetermines higher anxiety differentially in males and females. It seems to be determined by the blocking of aromatization in males, indicating that aromatized estradiol plays a prominent role in the organization of this behavior in males, while AR activity seems to be important in determining sexual dimorphism in anxiety‐like behaviors. Furthermore, our results demonstrate that exposure to, or even deficiency in, T during critical periods of development may have long‐lasting consequences, highlighting the importance of including both sexes in future studies. However, due to the fact that androgens and their metabolites can interact with a wide variety of receptors, it is challenging to definitively determine their effects. It is difficult to ascertain whether the effects of treatments with AR ligands, such as testosterone and DHT, are caused by their action at the AR or result from the activation of other receptors because of the quick conversion into androgens, estrogens, or other metabolites. Further research should be done on the actions of testosterone and how its metabolites affect different behaviors.

## Author Contributions

Each author has made substantial contributions to the work. B.C., P.C., and H.P. conceptualized the study, wrote the original draft, supervised the research, and reviewed and edited the manuscript. U.P., J.M.F.‐G., and R.G.‐Ú. conducted the investigation, performed the formal analysis, and reviewed and edited the manuscript. P.C. and H.P. administered the project, acquired funding, and supervised the research. All authors have read and agreed to the published version of the manuscript.

## Funding

The present work was supported by grants PSI2021‐115829GB‐I00 and IMIENS (PC/HP) from the Ministerio de Economía, Industria y Competitividad (, AEI, Spanish Government). It was also supported by a predoctoral fellowship (FPU23/04006) from the Spanish Ministry of Universities, a research grant from the Universidad Nacional de Educación a Distancia (UNED), and a Banco Santander predoctoral fellowship.

## Conflicts of Interest

The authors declare no conflicts of interest.

## Data Availability

The data presented in this study are available on request from the corresponding author.
